# The relationship between serum zinc level and preeclampsia

**Published:** 2010

**Authors:** Parvin Bahadoran, Manoush Zendehdel, Ahmad Movahedian, Roshanak Hasan Zahraee

**Affiliations:** * Department of Midwifery, School of Nursing and Midwifery, Isfahan University of Medical Sciences, Isfahan, Iran; ** Rasht, Iran; *** Associate Professor, Department of Biochemistry, School of Pharmacology, Isfahan University of Medical Sciences, Isfahan, Iran

**Keywords:** Serum zinc, risky pregnancy, preeclampsia, pregnancy outcomes, zinc supplement

## Abstract

**BACKGROUND:**

Preeclampsia is one of the commonest causes of prenatal and maternity related death in the world. Preeclampsia is caused by multiple factors and finding any factor related to this disorder can help on time prevention of this disease, which reduces the mortality of mothers and infants. Zinc deficiency is a possible risk factor for risky pregnancies and the results of studies on this subject are controversial. This study investigated the relationship between mothers’ serum zinc and risky pregnancies.

**METHODS:**

This was a case-control study on 48 normal pregnancies as controls and 48 preeclamptic pregnancies as case group. The women were studied in their third month of pregnancy. Simple random sampling was done based on inclusion and exclusion criteria. The two groups were matched in mothers’ age, pregnancy age, number of childbirth, and socioeconomic status. Data were collected by blood sampling and a questionnaire. Serum zinc level was assessed by atomic absorption spectrometry method. Data were analyzed using SPSS Software.

**RESULTS:**

The level of serum zinc in most women in both groups was under 50 mg/dl (62.5% in normal pregnancy group and 79.2% in preeclamptic group). There was no significant difference between the mean (SD) serum zinc concentration of the two groups (47.83 (12.72) for normal pregnancy and 43.66 (11.98) for preeclampsia). There was an association between serum zinc concentration and the severity of preeclampsia (p = 0.04, r = -0.12). We did not find any significant relation between serum zinc level and the following variables: mothers’ age (p = 0.15, r = -0.11), pregnancy age (p = 0.07, r = -0.24), and parity (p = 0.02, r = -0.39).

**CONCLUSIONS:**

The findings of this study showed that the assessment of serum zinc level does not have any clinical values for managing preeclampsia. However, based on the relationship between serum zinc concentration and the severity of preeclampsia in this study, we recommend assessment of serum zinc concentration as an index for predicting the severity of preeclampsia.

About 600,000 women die because of pregnancy and delivery complications every year in the world and about 50% of these cases are due to risky pregnancies.^1^ Preeclampsia, as a risky pregnancy, is the commonest cause of prenatal and maternity related death in the world.^2^ In the developing countries, women lost their lives due to preeclampsia every year and the risk of infants’ mortality in preeclampsia is 4 times higher than that in normal pregnancies.^2^ Preeclampsia is the third common cause of mothers’ mortality in the world^3^ and the second common cause of mother’s mortality in Iran; 18% of mothers’ mortality in Iran is due to preeclampsia.^4^ Diagnosis of preeclampsia is considered in women whose blood pressure increases to 90/140 mmHg for the first time after the 20^th^week of pregnancy while they have proteinuria.^3^

In spite of several-decade studies on preeclampsia, the cause is still unknown. Since preeclampsia is caused by multiple factors, finding any factor related to this disorder can help on time prevention and as a result can reduce the mortality of mothers and infants. Zinc deficiency, as a possible risk factor for preeclampsia, is questionable and the results of latest studies showed that lack of zinc causes increased lipid level and peroxidation, which suggest the possibility of the role of zinc deficiency in incidence of preeclampsia by increasing lipid peroxidation.^5^ Besides, the importance of micronutrients in preeclampsia was related to their biological roles, as cofactors of antioxidant enzymes.^6^

Studies conducted on the relationship between mothers’ serum zinc level and incidence of preeclampsia in different countries reported controversial results. Therefore, the existence of such relationship is not clear yet. Several studies showed a relationship between serum zinc deficiency and preeclampsia.^5^,^7^,^8^ But there are several studies that did not support this relationship.^9^,^10^ World Health Organization has set prevention and decrease of maternity and pregnancy related death in the priorities of health care programs for improving mothers and infants health.

It seems that finding the factors related to incidence of risky pregnancies is very effective for preventing risk factors and improving the health care method for mothers and infants. Zinc deficiency is a probable risk factor for occurrence of preeclampsia. Studies reported high prevalence of lack of zinc in Iran.^11^ Researchers recommended further studies on this field. Since the complications of preeclampsia especially in severe cases lead to short term and long term disabilities of mothers and infants and significantly affect their quality of life and as a result affect the health of family and society, this study was conducted to determine and compare the mothers’ serum zinc concentration in the two groups of normal pregnancy and preeclampsia and find the relationship between mothers’ serum zinc concentration and preeclampsia.

## Methods

This was an analytical case-control study. The study population included pregnant women in their third trimester of pregnancy, referred to emergency rooms of maternity or maternity clinics of Shahid Beheshti, Alzahra, Shariati and Asgariyeh hospitals in the city of Isfahan. The number of sample (with accuracy of 16 and reliability of 95) was estimated 96 women who were divided into two groups of control (normal pregnancy) and case (preeclampsia), 48 women in each group.

The inclusion criteria were as physically healthy, single pregnancy, pregnancy age of 31 to 40 weeks, willing to participate in the study, body mass index (BMI) between 18.5 to 29.99 kg/m^2^, and at least 4 kg weight gain until third trimester of pregnancy. The exclusion criteria included diagnosis of abnormal embryo in ultrasound, having chronic disease such as diabetes, chronic hypertension, chronic and severe disorders in kidneys, adrenal, liver, thyroid, parathyroid, and cardiovascular diseases, blood disorders, indigestion disorders, infections, malignant disorders, immune disorders, alcohol or drug addiction, smoking, severe stress during pregnancy, battered wives, infertility history, midwifery problems such as decolman placenta, placenta previa, consumption of anticancer and immunosuppression drugs, anticoagulants, aspirins, more than 60 mg iron, calcium, and medicines which contain zinc.

The sampling was done through simple random sampling method and data collection instruments included checklist and questionnaire. The validity of the instruments was determined by content validity. For the atomic absorption spectrophotometry device, reliability and accuracy were assured by one person doing all the tests in one specific laboratory. Other data collection instruments included a blood pressure measuring device, a tape measure, pregnancy care cards, records of infants’ birth weights, patients’ medical files, and necessary devices to test serum zinc level.

We explained the objectives and methodology of the study to two midwives to collect data. The sampling for case group (preeclampsia) was based on inclusive and exclusive criteria and for the controls, we also considered matching criteria. After informing the samples about study objectives and collecting their written permissions, the questionnaires were completed by face to face interview and their blood samples (5 cc) were collected. The subjects’ heights were measured by a tape measurement without shoes. Data of mothers’ weights at the beginning of pregnancy were recorded from their pregnancy care cards. To control falsifying factors, the two groups of case and control were matched for mothers’ age (maximum difference of 5 years), pregnancy age (maximum one week), and number of childbirths. Exclusion criteria also controlled some of the falsifying factors and the effects of other factors such as socioeconomic status (area of residence, mothers’ education and their husbands’, mothers’ career and their husbands’, family income, number of family members, and house ownership) were controlled by statistical methods. All tubes and head samplers were washed by acid to be clean of little elements. The complete blood samples were centrifuged for 10 minutes within 45 minutes and the serums were collected in a polyethylene covered tube already washed well with acid. Samples were frozen in -20 °C until being analyzed. Sampling and follow-up to complete infant data continued for 8 months, from September 2007 until April 2008. After sampling finished, the samples were transferred in a cold box to the biochemistry unit in Faculty of Pharmacology of Isfahan University of Medical Sciences. The serum zinc was measured by atomic absorption spectrophotometry.

To analyze the data, descriptive statistical methods (frequency, mean and standard deviation) and inferential statistical methods (Pearson’s correlation coefficient, t-test, chi square and ANOVA) were used via SPSS software.

The ethical committee of Isfahan University of Medical Sciences approved the study.

## Results

Most subjects in both groups were in the age range of 20-30 years (81.3% of control and 72.9% of case group). Most women in the study (56.3% in each group) had no history of childbirth and the pregnancy age at the time of sampling was 36-40 weeks for most subjects in both groups. The mothers’ mean (SD) age was 27.06(4.67) years for the normal pregnancy group and 27.56(4.87) years for the preeclamptic group. The mean (SD) age of pregnancy at the time of sampling was 36.36(2.60) years for the normal pregnancy group and 36.34(2.63) years for the preeclamptic group. As it was expected, the results of t-test showed that the two groups were matched in mothers’ age, number of childbirth, and pregnancy age at the time of sampling.

The serum zinc level in most subjects in both groups (62.5% for normal group and 79.2% for preeclamptic group) was under 50 mg/dl. The mean (SD) serum zinc concentration was 47.83(12.72) for normal pregnancy group and 43.66(11.98) for preeclamptic group and the t-test showed no significant difference between the two groups (p = 0.102). The mean (SD) serum zinc concentration in patients with severe preeclampsia 10.24(37.50) was lower than that in patients with mild preeclampsia 12.03(44.90); also in patients with mild preeclampsia it was lower than that in normal pregnancy group. ANOVA showed a significant difference between the mean serum zinc level of the severe preeclamptic patients and normal pregnancy group as well as between mild and severe preeclamptic subgroups (p = 0.04). But, there was no significant difference between patients with mild preeclampsia and those with normal pregnancies on mean serum zinc level (p = 0.24). The results of Pearson’s correlation coefficient showed no significant relation between serum zinc and the following variables: mothers’ age (p = 0.15, r = -0.11), pregnancy age (p = 0.07, r = -0.24), parity (p=0.02, r = -0.39), and infants’ weights (p = 0.10, r = 0.13). There was no significant difference between the two groups on socio-economic status.

## Discussion

The results of this study showed that the serum zinc level in preeclampsia group was lower than that in normal pregnancy group, but the difference was not statistically significant. This is supported by the results of some other studies. In Atamer et al study the mean (SD) serum zinc in preeclampsia with the amount of 18(2.79) was lower than that in normal pregnancies with the amount of 19.9(6.108), but the difference was not significant.^9^ In the study of Adam et al also the mean serum zinc in preeclampsia with the amount of 4.7(31.3) was lower than that in normal pregnancy with the amount of 4.4(34.1). As we can see, the mean serum zinc level in both groups of Adam’s study was lower than that in our study, but the difference in serum zinc level was not significant between the two groups.^10^ Controversial to the results of the present study, in a study by Ilhan et al the mean serum zinc level in normal pregnancy group and preeclampsia was 23.24 (125.19) and 28.93 (82.94), respectively and the difference was statistically significant.^7^ The mean serum zinc level in both groups of Ilhan’s study was significantly high. In a study by Ikgoz et al also the mean of serum zinc level in preeclampsia was significantly different from that of normal pregnancy group.^8^ However, in the study of Harma et al in Turkey the results showed that the mean plasma zinc level in preeclampsia group was significantly higher than that in normal pregnancy group.^12^ In the study of Diaz et al also the level of serum zinc in preeclampsia was higher than that in normal pregnancy (110 vs. 99 mg/dl), but the difference was not significant.^13^

Researchers believe that consumption of zinc by infant and pregnancy products increases the blood volume and decreases the serum albumin level and causes possible hormonal changes,^14^ as well as zinc shift from plasma to red corpuscle can be the possible cause of serum zinc decrease in pregnant women.^15^ The low level of serum zinc in the present study compared to other studies can be related to geographical differences and different nutrition and foods. These causes should be investigated and prevented. Because of the significant low levels of serum zinc in both groups of the present study as well as other studies in Iran,^16^,^17^ it is recommended to design a program to investigate the condition of zinc level in women’s body during pregnancy and provide zinc supplements for those who need. Also, it is recommended to increase the knowledge and attitude of pregnant women as a usual pregnancy care service throughout the country. The serum zinc deficiency among pregnant women in Iran can be related to before pregnancy and even during childhood, which has been ignored. Therefore, considering the fact that zinc deficiency will put young women and future mothers at risk of growth disorders and cause economic, social, clinical and health complications, in order to have a healthy generation it is recommended that by the cooperation of health ministry and education ministry, in a national screen program, actively seek the zinc deficiency in teenagers in guidance schools and high schools around the country and provide treatment supplements in safe dosage for those who need.

Different study designs, limitation of statistical methods in studies on small samples, different techniques of analysis and also different population characteristics such as race, ethnicity, culture, eating habits and geographical regions are possible explanations for the different results of this study with other studies. One of the interesting findings of this study was the relationship between serum zinc concentration and the severity of preeclampsia. Since the critical outcomes of pregnancy in women with severe preeclampsia and their infants are worse than mild preeclampsia cases, it is very important to determine and predict severe preeclampsia cases. Of course, there are few studies in Iran and other countries on predicting factors of severe preeclampsia and we were not able to find any paper on the relationship between the serum zinc concentration and the severity of preeclampsia. However, in a study by Dawson et al in 1999, the results showed that the level of liquid amniotic zinc is associated with severe preeclampsia.^18^ Considering the findings of this study, it is clear that by measuring the serum zinc concentration, we can predict the risky outcomes of preeclampsia that is severe preeclampsia, even though this measurement might not be a good index for mild cases of preeclampsia. Further studies are needed to investigate the measurement of serum zinc concentration as a test to predict the incidence of severe preeclampsia in women with risk factors or symptoms of preeclampsia.

The authors declare no conflict of interest in this study.
